# Transcriptomics and Gene Family Identification of Cell Wall-Related Differentially Expressed Genes Reveal *MaXTH32.5* Involved in Fruit Firmness During Banana Ripening

**DOI:** 10.3390/plants14243810

**Published:** 2025-12-14

**Authors:** Fengjie Yang, Kui Wan, Xiaoli Kang, Wanting Zhong, Jiasi Lv, Yiyao Lin, Jialing Wang, Zhongxiong Lai, Bin Liao, Yuling Lin

**Affiliations:** Institute of Horticultural Biotechnology, Fujian Agriculture and Forestry University, Fuzhou 350002, China

**Keywords:** *Musa* spp., *Xyloglucan endotransglucosylase/hydrolase* (*XTH*), ripening, firmness

## Abstract

Banana (*Musa* spp.) is a typical climacteric fruit. *Xyloglucan endotransglucosylase/hydrolase* (*XTH*) is a key factor regulating plant cell wall dynamic remodeling and participates in fruit ripening. To clarify the core physiological traits of banana ripening, four ripening stages of banana cultivar (*Musa* AAA ‘Minai No. 1’) fruits in the fully green stage (S1), green-yellow stage (S2), fully yellow stage (S3), and yellow with brown spots stage (S4) were used in this study’s experimental materials, to examine dynamic changes in key physiological–biochemical properties. The results showed that fruit firmness decreased continuously, starch content first increased then decreased, and soluble protein and total soluble solids (TSS) accumulated gradually during the ripening stages of banana fruits. Transcriptome analysis of the four stages found that there were 14,315 differentially expressed genes (DEGs) in S1 versus S4, the GO enrichment pathway is enriched in “protein dephosphorylation”, and the KEGG enrichment pathway is enriched in the “Protein processing in endoplasmic reticulum” and “Ubiquitin mediated proteolysis” pathways. The fruit ripening process involves the processing of numerous proteins. The heatmap revealed that *MaXTH32.5* was significantly up-regulated during banana ripening and the result of RT-qPCR is consistent with the transcriptome data. A total of 989 *XTH* members across 16 *Musa* varieties of the XTH gene family were further identified. Among them, MaXTH32.5 localized at the chloroplast, and transient overexpression of *MaXTH32.5* significantly reduced banana fruit firmness and may be involved in regulating ripening in banana fruits. This study indicated that the differential expression of XTH gene family members may regulate ripening-related processes in banana and *MaXTH32.5* as a key candidate, providing insights into banana ripening mechanisms and a foundation for subsequent *Musa XTH* research.

## 1. Introduction

Non-pectinolytic enzymes such as endo-1, 4-β-glucanases (EGs) and xyloglucan endotransglycosylases (XTHs) can induce fruit softening through the modification of cellulose [1]. Among them, xyloglucan is a component of hemicellulose in the plant cell wall, thereby providing strength to the cell wall during the growth period and also restricting the expansion and softening of the cell wall [2,3]. *XTH* catalyzes the non-hydrolytic cleavage and rearrangement of xyloglucan chains through the activity of xyloglucan endotransferase (*XET*) or directly through the activity of xyloglucan endohydrolase (*XEH*) [4]. Currently, research on XTH gene family identification mainly focuses on *Arabidopsis thaliana* [5] and *Oryza sativa* [6].

Banana is an evergreen tropical and subtropical monocotyledonous herbaceous plant belonging to the genus *Musa* of the family Musaceae in the order Zingiberales. As the fruit with the largest fresh fruit trade volume in the world, it has a high yield and is rich in fiber, vitamins, proteins, various minerals, and carbohydrates. It serves as a food source and staple food for more than 400 million people around the world [7]. According to statistics, approximately 33% of vegetables and fruits globally are wasted and lost after harvesting each year due to their short shelf life [8]. As a typical climacteric fruit, bananas have a very short shelf life. The main determinant affecting their commercial value and shelf life is fruit softening, which is mainly caused by the degradation of the cell wall [9]. Recent studies have shown that *XTH* plays vital roles during fruit ripening. The MdWRKY31-*MdNAC7* regulatory network orchestrates fruit softening by modulating the cell wall-modifying enzyme *MdXTH2* in response to ethylene signaling [10]; transcriptome analysis found that the expression levels of the XTH gene family were highly up-regulated during the ripening process of banana fruits [11]; through integrated transcriptomic, proteomic, and metabolomic analysis, XTHs were identified as the core genes for cell wall modification during the natural ripening of banana peels [12]. The above results imply that XTHs regulate ripening by affecting fruit firmness through cell wall metabolism in banana. With the release of banana genomes such as A, B, and S/T [13,14,15,16], an increasing amount of genomic data has become available. In previous studies, using only the A reference genome, 53 members of the XTH gene family were identified [17]. However, pan-genome family identification has not yet been conducted, and it remains unclear whether additional members are involved in banana ripening.

Herein, we used four ripening stages of banana cultivar (*Musa* AAA ‘Minai No. 1’) fruits in the fully green stage (S1), green-yellow stage (S2), fully yellow stage (S3), and yellow with brown spots stage (S4) as experimental materials for the measurement of firmness, starch content, soluble protein content, and soluble sugar content. By performing transcriptome sequencing on these four stages, the differentially highly expressed MaXTHs were screened out. A pan-genome identification of the XTH gene family was carried out, and the basic physical and chemical properties, chromosomal localization, phylogenetic relationships, gene structures, conserved protein domains, and cis-acting elements in the promoters of each member were analyzed. Additionally, the RT-qPCR technique was used to detect the expression levels of the XTH gene family in the four ripening stages of bananas. MaXTH32.5 localized to the chloroplast and it was found that transient expression of *XTH32.5* indicates a close relationship with fruit softening. The aim is to understand the biological functions and expression patterns of the XTH gene family in bananas, providing a theoretical reference for the screening and analysis of functional genes.

## 2. Results

### 2.1. Analysis of Changes in Physiological Indicators During Banana Fruit Ripening

**Figure 1:**
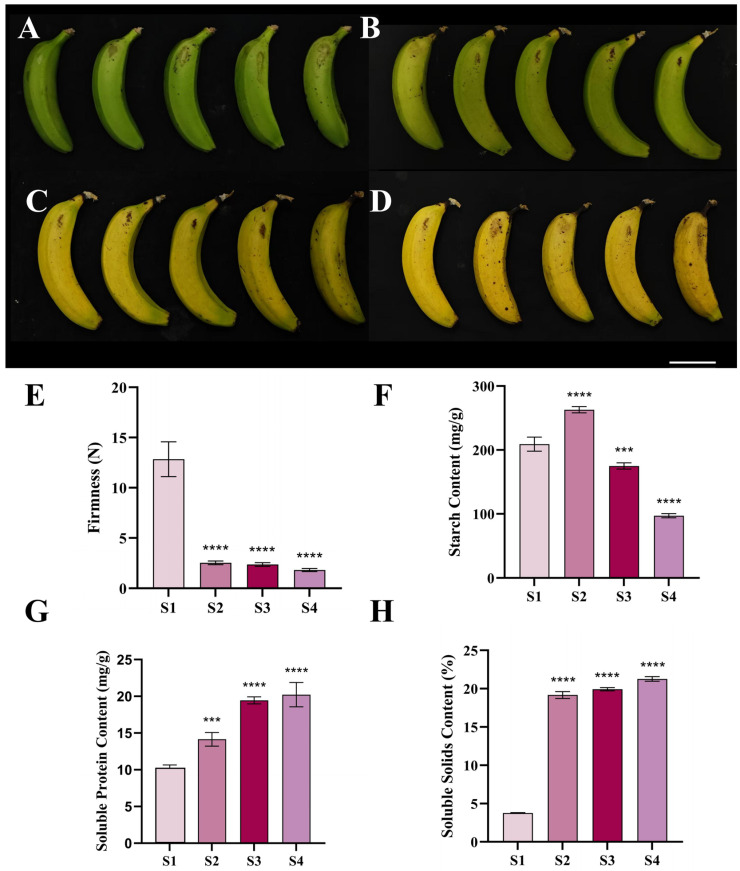
Determination of indicators for banana fruits in four ripening stages. (**A**) S1. (**B**) S2. (**C**) S3. (**D**) S4. Scale bar, 3 cm. (**E**) Firmness determination. (**F**) Starch content determination. (**G**) Soluble protein content determination. (**H**) Soluble solid content determination (*** *p* < 0.001, **** *p* < 0.0001).

In this study, the changes in the firmness, starch content, soluble protein content, and soluble solid content of the fruits of banana in four ripening stages (S1: fully green, S2: green-yellow, S3: fully yellow, and S4: yellow with brown spots) were measured. The results are shown in Figure 1A–D. During the ripening process of banana fruits, the fruit firmness showed a continuous downward trend (Figure 1E). The firmness value in the fully ripe stage (S4) decreased by 85.81% compared with that in the fully green stage (S1), indicating that the cell wall structure underwent significant deconstruction during the ripening process. The starch content showed dynamic fluctuations of first increasing and then decreasing (Figure 1F). It increased significantly by 25.70% in the mid-green ripening stage (S2) compared with the S1 stage, and then decreased by 63.04% from the S2 stage to the S4 stage. The soluble protein content showed a steady upward trend (Figure 1G). The cumulative increase in the S4 stage was 97.52% compared with the S1 stage. The soluble solid content gradually increased during the ripening process, and it increased by 4.65 times in the S4 stage compared with the S1 stage (Figure 1H).

### 2.2. Transcriptome Reveals the Differential Expression of MaXTH During Fruit Ripening

The above results indicated that changes such as firmness and soluble solid content were closely associated with the ripening of fruits. To investigate the biological roles of genes, we conducted a transcriptomic analysis of the fruit ripening using RNA-seq. A total of 12 cDNA libraries were constructed for the fruits (S1, S2, S3, and S4). We further screened the significantly differentially expressed genes (DEGs) with |log_2_ fold change| ≥ 1 and false discovery rate (FDR) < 0.05 between these three pairwise comparisons as follows: S1 vs. S2, S1 vs. S3, and S1 vs. S4 comparisons. Across the three pairwise comparisons of banana ripening stages (S1 vs. S2, S1 vs. S3, and S1 vs. S4), the number of differentially expressed genes (DEGs) and their expression trends were summarized as follows: a total of 13,258 DEGs were identified, including 9248 up-regulated and 4010 down-regulated genes in S1 vs. S2; 13,543 DEGs, with 9264 up-regulated and 4279 down-regulated in S1 vs. S3; and the highest number of DEGs was detected (14,315), consisting of 9412 up-regulated and 4903 down-regulated genes in S1 vs. S4 (Supplementary Figure S1).

**Figure 2:**
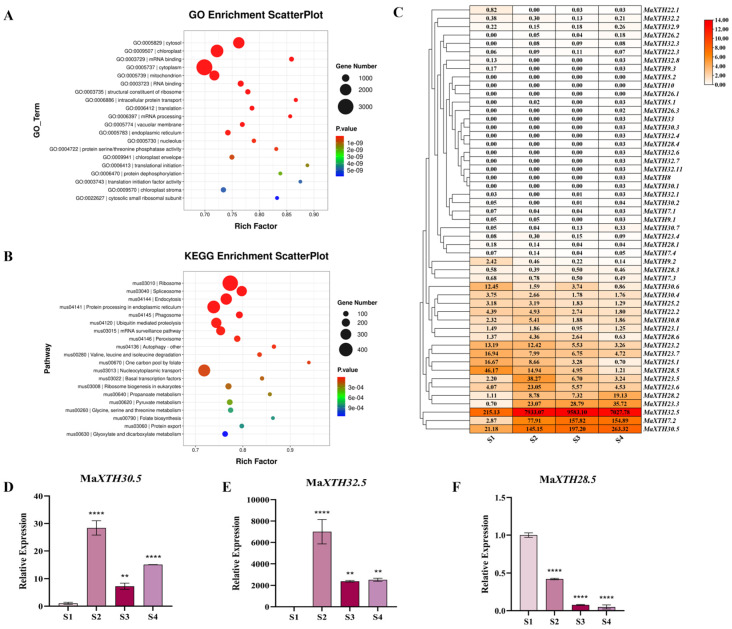
Analysis of transcriptome and RT-qPCR in different banana ripening stages. (**A**) GO enrichment bubble plot. (**B**) KEGG enrichment bubble plot. (**C**) Expression profiles of MaXTHs in different banana ripening stages. (**D**) Expression analysis of *MaXTH30.5* in four ripening stages of bananas. (**E**) Expression analysis of *MaXTH32.5* in four ripening stages of bananas. (**F**) Expression analysis of *MaXTH28.5* in four ripening stages of bananas (** *p* < 0.01, **** *p* < 0.0001).

Across the comparisons of banana ripening stages (S1 vs. S4), the GO enrichment bubble pattern reveals that the GO enrichment pathway is enriched in the “protein dephosphorylation” and “vacuolar membrane” pathways (Figure 2A). The results of the KEGG hierarchy bar chart show that differentially expressed genes are enriched in the “Protein processing in endoplasmic reticulum” and “Ubiquitin mediated proteolysis” pathways (Figure 2B). The fruit ripening process involves the synthesis and processing of numerous proteins. The endoplasmic reticulum is the site of protein synthesis and primary processing. This pathway may be involved in regulating the correct folding, modification, and other processes of related proteins in the fruit, affecting fruit ripening. The ubiquitin-proteasome system is involved in regulating intracellular protein degradation.

In order to screen the key XTHs related to banana fruit ripening, we analyzed the expression levels of 51 MaXTHs identified from the A genome. The heatmap shows that five MaXTHs (*MaXTH32.5*, *MaXTH23.3*, *MaXTH30.5*, *MaXTH7.2*, and *MaXTH28.2*) were significantly up-regulated, and four MaXTHs (*MaXTH23.2*, *MaXTH23.7*, *MaXTH28.5*, and *MaXTH25.1*) were down-regulated during fruit ripening.

We selected seven MaXTHs (*MaXTH23.3*, *MaXTH28.5*, *MaXTH23.6*, *MaXTH7.2*, *MaXTH23.5*, *MaXTH30.5*, and *MaXTH32.5*) from the transcriptome data for quantitative analysis using the RT-qPCR technique. The results showed that during the ripening process of banana fruits, *MaXTH32.5* had the highest average expression level in each stage, followed by *MaXTH30.5*, and the expression level of *MaXTH32.5* increased by 6841.46 -fold in S2. The opposing effect of *MaXTH28.5* may play a role in maintaining dynamic balance. This is consistent with the transcriptome data. The other several genes may have played synergistic roles (Supplementary Figure S2).

### 2.3. Identification and Characteristics of XTH32.5s

**Figure 3:**
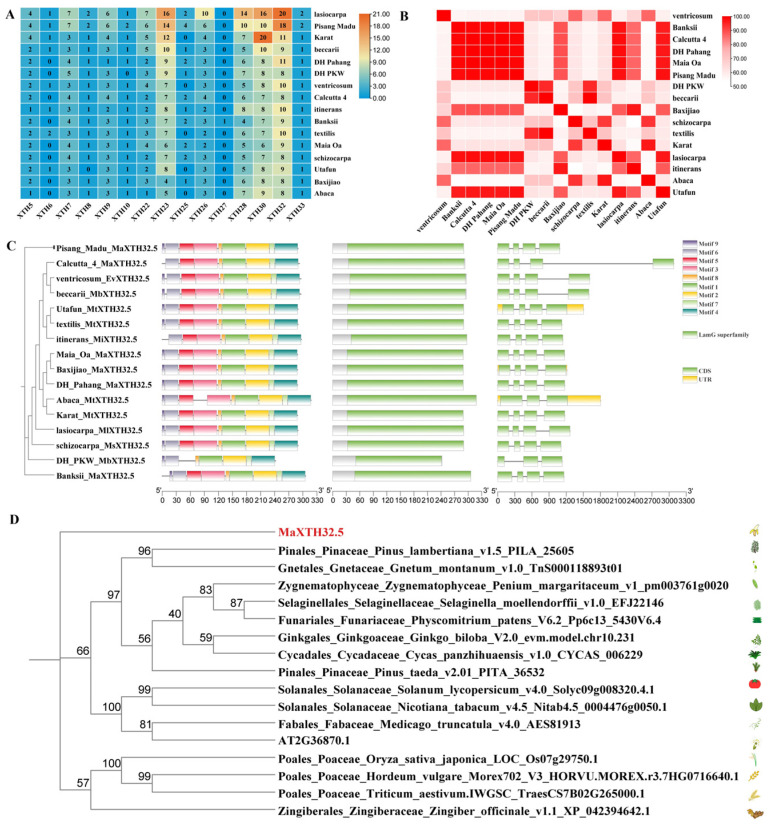
Identification and characteristics of XTH32.5s. (**A**) Number of members of XTHs in different genomes. (**B**) Matrix diagram. (**C**) Phylogenetic tree, conserved motifs, conserved domains, and gene structure of XTH32.5. (**D**) Phylogenetic evolutionary tree of MaXTH32.5 and XTH proteins from other species.

According to the above results, *XTH* may play a role in the banana ripening process. However, the current gene family identification is not yet systematic (only in *Musa* acuminata DH Pahang). Therefore, the identification of the XTH gene family was carried out based on 16 *Musa* species accessions. The number distribution of XTHS homologs among different *Musa* species accessions is presented in Figure 3 (Figure 3A). A total of 989 members of the XTH gene family were identified in the 16 *Musa* species accessions. They were named referring to the naming method of the XTH gene family members in *Arabidopsis thaliana*. Among them, XTH32 (183 members), XTH30 (164 members), XTH23 (145 members), and XTH28 (117 members) have a relatively large number in different *Musa* species accessions. To study the evolutionary relationships among the members of the XTH gene family in different *Musa* species accessions, combining with the classification system of the XTH family in *Arabidopsis thaliana*, the XTHs of the 16 *Musa* species accessions were divided into five major groups (Supplementary Figure S3). Among them, XTH32, XTH30, and XTH33 are all located on independent branches, belonging to I/IIB, IIIB, and the early diverging group, respectively. This indicates that the evolutionary relationships of these genes with other genes are relatively distant, and they may have specific functions.

This heatmap (Figure 3B) illustrates the similarity of the MaXTH32.5 across various species within the *Musa* genus. The rows and columns list different *Musa* species accessions (such as ventricosum, Banksii, Calcutta 4, and DH Pahang). The color gradient, ranging from light pink to dark red, represents the level of MaXTH32.5-related signal (this “signal” refers to the conservation or variability of the gene sequence). Darker red indicates a higher level. From the heatmap, we can observe that species like Banksii, Calcutta 4, DH Pahang, Maia Oa, Pisang Madu, Abaca, and Utafun showed relatively high levels of the MaXTH32.5-associated signal, as evidenced by the prominent dark red squares. In contrast, other species exhibit lower levels, with lighter pink or even white (indicating the lowest level) in their corresponding squares. This pattern suggests potential variations in the role or presence of MaXTH32.5 across different *Musa* species accessions, which could be related to evolutionary adaptations, functional diversifications, or specific physiological processes in each species.

To further understand the conservation and diversity of XTH32.5 members in different *Musa* species accessions, analyses of gene structure, protein-conserved motifs, and conserved domains were carried out. In XTH32.5 proteins, motif1, motif2, motif4, motif6, motif7, and motif8 (6 motifs) are commonly present, and motif10 is generally absent. Motif9 is absent in Calcutta_4_MaXTH32.5 and itinerans_MiXTH32.5, and motif3 and motif5 are only absent in DH_PKW_MbXTH32.5.

Analysis of the conserved domains of XTH32.5 proteins was carried out using the CDD website (Figure 3C). Gene structure analysis shows that most XTH32.5 members contain 4 exons and 3 introns. Additionally, DH_PKW_MbXTH32.5 contains 3 exons and 2 introns; Abaca_MtXTH32.5 has 5 exons and 4 introns; and Utafun_MtXTH32.5 and Baxijiao_MaXTH32.5 have 6 exons and 5 introns.

The amino acid sequences of MaXTH32.5 were aligned with those of the XTH32.5 with the highest homology from 17 species. Then, a phylogenetic tree was constructed. The results showed that MaXTH32.5 was located on independent branches, and had low homology with the XTH proteins of the 17 species (Figure 3D).

### 2.4. Chromosomal Distribution, Synteny, Cis-Acting Elements, and Sequence Differences of MaXTH32.5

**Figure 4:**
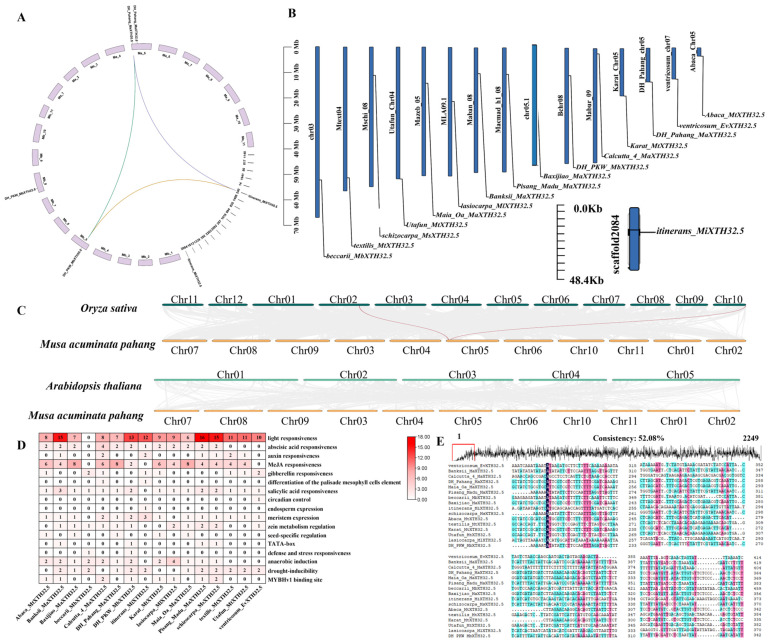
Chromosomal distribution, synteny, *cis*-acting elements, and sequence differences of *MaXTH32.5*. (**A**) Synteny analysis of *XTH* in *Musa*. (**B**) Chromosomal localization analysis of *XTH32.5*. (**C**) Synteny analysis of *XTH* between *Musa* acuminata pahang and two model plants (*Arabidopsis thaliana* and *Oryza sativa*). (**D**) Analysis of *cis*-acting elements in the *XTH32.5* promoter. (**E**) Analysis of promoter sequence.

The *Musa* species accessions of the A (DH Pahang) genome, B (DH PKW) genome, and i (tinerans) genome were selected as representatives to analyze the interspecific collinearity of XTH32.5s. The intraspecific collinearity analysis shows that XTH30.5 of the A genome has collinearity with both XTH30.5 of the B genome and XTH30.5 of the i genome (Figure 4A). Chromosomal localization shows (Figure 4B) that among the 16 *Musa* species accessions, XTH32.5s are located on separate chromosomes. The chromosome of ‘itinerans’ is relatively short. For XTH32.5, 31.25% of the genes are distributed on chromosome 5 of their respective genomes, 25% are on chromosome 8, and the remaining ones are distributed on chromosomes 3, 4, 7, 9, and scaffold2084. The results of the collinearity analysis between the A genome of the *Musa* genus and *Arabidopsis thaliana* and *Oryza sativa* (Figure 4C) show that there are two pairs of collinear relationships between the XTH members of the A genome and *Oryza sativa*, while there is no collinearity with *Arabidopsis thaliana*. This indicates that the XTHs of the *Musa* genus have a closer relationship with those of *Oryza sativa*.

A systematic analysis of the *cis*-acting elements in the promoter regions of XTH32.5s in different *Musa* species accessions was carried out using PlantCARE. The results showed that in addition to a large number of light-responsive elements, the promoters also contain various elements that respond to hormones, growth and development, and stress. For the promoters of XTH32.5 members in different *Musa* species accessions, a total of 399 *cis*-acting elements were identified. Among them,’ventricosum’ contains the largest number of *cis*-acting elements (37), followed by ‘Abaca’ (36) and ‘Calcutta 4’ (35), and the number of elements in the remaining *Musa* species accessions ranges from 14 to 33. Each *XTH32* in different *Musa* species accessions contains at least one plant hormone-responsive element (Figure 4D). Among them, 87.5% contain abscisic acid-responsive elements and methyl jasmonate-responsive elements, 50% contain auxin-responsive elements, 31.25% contain gibberellin-responsive elements, and 18.75% contain salicylic acid-responsive elements. In addition, ‘itinerans’ also contains one element responsive to palisade mesophyll cell differentiation. A total of 93.75% of the XTH32.5s in *Musa* species accessions contain elements responsive to growth and development. Among them, 62.5% contain elements responsive to meristem expression, 50% contain elements responsive to endosperm expression, 43.75% contain the core element TATA-box, 25% contain elements responsive to circadian rhythm, and 18.75% contain elements responsive to zein metabolism and seed specificity. There is at least one element responsive to stress. A total of 87.5% of the XTH32.5s in *Musa* species accessions contain elements responsive to anaerobic induction, 50% contain MYB binding sites for drought stress, 31.25% contain elements responsive to wounding, and 18.75% contain elements responsive to defense, stress, and low temperature. In addition, the XTH32.5s of ‘Maia Oa’ and ‘Pisang Madu’ also contain elements related to elicitor-mediated responses. By analyzing the sequence differences of the *XTH32.5* promoter among different *Musa* species accessions, it was found that the identity of *XTH32.5* among different *Musa* species accessions was 52.08% (Figure 4E). This result indicates that their promoters have large differences, which may be caused by the individual differences among different cultivars.

### 2.5. MaXTH32.5 Localized to the Chloroplast and May Be Involved in Regulating Firmness in Banana Fruits

**Figure 5:**
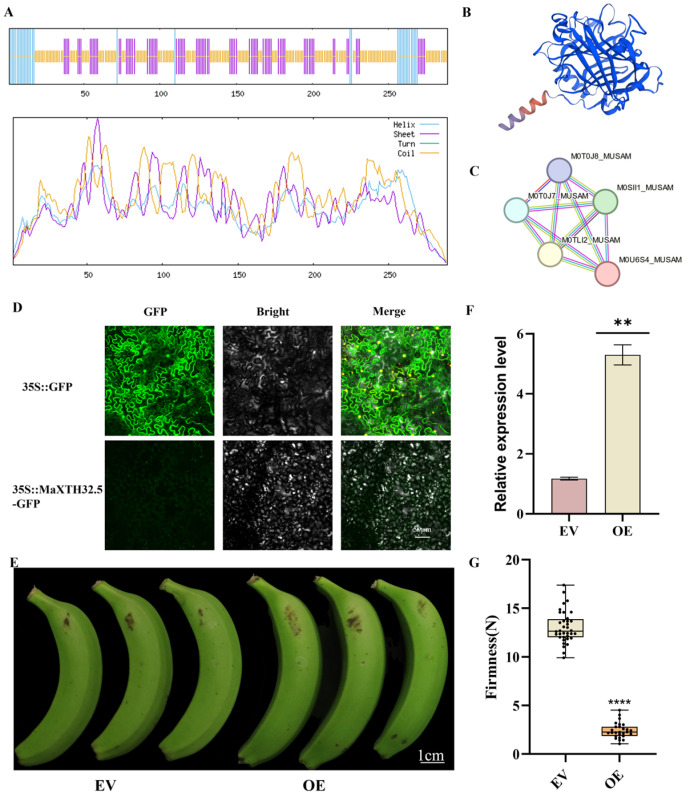
Functional characterization of *MaXTH32.5* in banana. (**A**) Predicted secondary structure diagram of MaXTH32.5 protein. (**B**) Tertiary structure prediction of XTH32.5. (**C**) Protein interaction network of XTH32.5. (**D**) Subcellular localization analysis of MaXTH32.5 protein. Scale bar, 50 μm. (**E**) Fruit phenotype. (**F**) MaXTH32.5 transient transformation fruit relative expression. (**G**) Firmness of EV (Empty vector) and (OE overexpression) fruit surfaces. Scale bar, 1 cm (** *p* < 0.01, **** *p* < 0.0001).

To further understand the structural characteristics of MaXTH32.5 proteins, the Sompa and Swiss model tools were used to predict their secondary and tertiary structures, and the String software was employed to predict their protein–protein interaction relationships. The structure prediction of the MaXTH32.5 protein indicated that most of it was random coils (58.48%), 29.07% were extended strands, and 12.46% were α-helix structures, which was consistent with the tertiary structure prediction (Figure 5A,B). The prediction of the protein regulatory network showed that MaXTH32.5 proteins interacted with M0TLI2_MUSAM (60.4%), M0SII1_MUSAM (42.8%), M0T0J7_MUSAM (40.9%), and M0T0J8_MUSAM (40.9%). Among them, the M0TLI2_MUSAM protein can participate in transcriptional activation through interactions with the TREX-2 and SAGA complexes (Figure 5C).

Fluorescence signals in cells were observed using a laser confocal microscope. The results showed that the fluorescent signal of MaXTH32.5-GFP fusion protein was mainly enriched in chloroplasts, indicating that MaXTH32.5 protein is localized in chloroplasts. In contrast, in tobacco cells transiently expressing the pCAMBIA1302 empty vector, the GFP signal was evenly distributed in all regions of the cells. These results confirm that MaXTH32.5 has a typical chloroplast localization characteristic, which is consistent with its biological functional properties (Figure 5D).

To investigate the function of *MaXTH32.5* in banana, *MaXTH32.5*-OE was obtained through banana transformation (Figure 5E). Quantitative real-time polymerase chain reaction (RT-qPCR) analysis revealed that the relative expression level of *MaXTH32.5* in the overexpression group was significantly higher than that in the Empty Vector (EV), showing a 4-fold up-regulation (Figure 5F). Furthermore, to investigate the function of *MaXTH32.5* in the banana ripening process, the fruit firmness of banana fruits with *MaXTH32.5* overexpression was measured (Figure 5G). The results indicated that compared with the empty vector control group, the banana fruits overexpressing *MaXTH32.5* exhibited promoted softening. *MaXTH32.5* can accelerate cell wall degradation, thereby leading to fruit softening. In conclusion, the Agrobacterium-mediated overexpression of *MaXTH32.5* in transformed banana fruits affects the fruit ripening rate, which provides an important basis for in-depth research on the function of *MaXTH32.5* in banana fruit development.

## 3. Discussion

*XTH* is a key regulatory factor in plant cell wall remodeling. By dynamically regulating the xyloglucan network in the cell wall, it participates in important physiological processes such as cell elongation, organ development, and stress response. In this study, a systematic identification of the XTH gene family in the genus *Musa* was carried out through pan-genome analysis, suggesting it may play a certain role in the fruit ripening process.

During the ripening process of ‘*Musa* AAA’ fruits, the firmness decreased significantly (the firmness at stage S4 was 85.81% lower than that at stage S1), which is closely related to the degradation of cell wall polysaccharides (such as cellulose and xyloglucan) in banana fruits [18]. Their study found that structural polysaccharide breakdown directly leads to fruit softening, providing cross-validation for the “polysaccharide degradation-firmness loss” mechanism in our results. The starch content first increased and then decreased. It increased by 25.70% at stage S2 compared with stage S1, and decreased by 63.04% at stage S4 compared with stage S2. It is speculated that at stage S2, the regulation of energy storage may be achieved through the transient activation of starch synthase, and the high expression of amylase in the later stage dominates the degradation process [19,20]. These basic findings are consistent with the core mechanism of banana starch “synthesis-degradation” and enzyme regulation, but supplements the quantitative data of the full ripening stage and metabolic correlation across the complete cycle for the specific ‘*Musa* AAA’ cultivar. The content of soluble proteins continuously accumulates during the ripening process of bananas (the content at stage S4 increased by 97.52% compared with that at stage S1), which may be related to the dynamic expression of relevant genes during the ripening process of banana fruits. For example, PHO1 (phosphatase) plays a key role in the metabolic conversion of starch to sugar, and the up-regulation of its activity may indirectly drive the activation of the pathways related to protein synthesis [3]. The content of soluble solids in banana fruits gradually increased during the four ripening stages, which is consistent with the theory that the degradation of starch drives the accumulation of sugars during the ripening process of bananas [21].

The transcriptome analysis of XTHs in different ripening stages of bananas showed that the expressions of different MaXTHs varied greatly. *XTH* has also been identified as a key ripening-related gene from different omics approaches [12]. The “protein processing and degradation” pathway is enriched, which is consistent in the transcriptome study on ‘Fen Jiao’ [22]. This study pointed out that during the ripening process, fruits need to remove senescence-related proteins and activate key ripening enzymes (such as cell wall-degrading enzymes) through protein degradation. Furthermore, this study confirms the significance of this pathway in the S1–S4 stages, indicating that “protein turnover” is a conserved pathway for banana ripening regulation. The results of this study provide a new cultivar case to support the universality of this pathway. Most existing studies focus on the role of *XTH* in the middle and late stages of fruit ripening. The expression levels of *PavXTH14* and *PavXTH15* reach their peak during the full ripening stage of sweet cherry fruits [23], whereas in this study, the expression peak of *MaXTH32.5* also appears in the full ripening stage. This expression profile reflected the spatiotemporal specificity of the regional modification of the cell wall [24], indicating that different members of the XTH family may play different biological functions during the ripening stages of bananas.

In this study, a total of 989 XTHs were identified in 16 species of the Musa genus. This difference in spatial distribution may be related to their functions. Analyses of gene structure and protein motif composition indicated that they were highly conserved. Analysis of their conserved domains revealed that, unlike the Glyco_hydro_16 and XET_C conserved domains contained in XTH of most species [25,26,27], it has a specific EXDXE motif and may be the catalytic site for XET and XEH activities [28]. The MaXTH32.5 with the GFP tag was introduced into tobacco leaf cells to determine its subcellular localization, and the results showed that it localized to chloroplasts. Previous studies have revealed that chloroplast signaling pathways play a key role in the initiation of fruit ripening [29]. Furthermore, transient transformation of MaXTH32.5 affects banana fruit ripening. In contrast, overexpression of *MdXTH2* can increase the fruit firmness of apples and tomatoes and delay fruit ripening [30].

## 4. Materials and Methods

### 4.1. Plant Materials

The tested material was banana (*Musa* AAA ‘Minai No. 1’), independently bred by the Institute of Horticultural Biotechnology, Fujian Agriculture and Forestry University, and grown in the university’s experimental orchard under natural conditions. Fruits were divided into four ripening stages based on peel color and firmness: S1 (fully green, hardness > 15 N), S2 (30–50% yellow, hardness 10–15 N), S3 (fully yellow, hardness 5–10 N), and S4 (yellow with 20–30% brown spots, hardness < 5 N). After cutting bunches into individual fingers, sterile filter paper was used for 30 min surface juice drainage in a laminar flow hood, followed by selection of fruits with consistent color, uniform size (18–22 cm in length, 3.5–4.5 cm in diameter), and no diseases, pests, or mechanical damage; three biological replicates (10 fingers each) were set for each stage, with middle sections of the peel (2 mm thick) and pulp (1 cm^3^) sampled, cut into small pieces, and immediately frozen in liquid nitrogen before storage at −80 °C. For transient transformation, S1-stage fruits were used and incubated in a growth chamber (25 °C, 70% relative humidity, dark) for 48 h post transformation, and 1 cm^3^ pulp tissue from the transformation site (three biological replicates, 3 fruits each) was collected, frozen in liquid nitrogen, and stored at −80 °C for subsequent experiments; all sampling tools were sterilized with 75% ethanol and flame to ensure aseptic conditions.

### 4.2. Determination of the Firmness and Other Physiological Indices of Banana Fruits

The firmness of banana pulp was measured using a firmness tester (GY-4). The undamaged area in the middle part of the fruit was selected for the test. Three samples were randomly selected for repetition in each ripening stage, and four different parts were selected from each banana. The banana was peeled with scissors, and then the firmness value of the banana pulp was measured. After grinding 20 g of banana pulp, it was centrifuged at 5000 r/min for 10 min at 20 °C. The supernatant was taken and measured using a refractometer. Referring to the instructions of each kit, the starch content and soluble protein content of the banana pulp were determined. The kits were all provided by Beijing Solarbio Science & Technology Co., Ltd. (Beijing, China). The obtained data were analyzed by one-way analysis of variance (ANOVA) and significance of difference (*p* < 0.05) using GraphPad Prism9.5 software, and bar charts were drawn using this software.

### 4.3. Transcriptome Sequencing, Assembly, Functional Annotation, and Heatmap

Transcriptome sequencing was carried out using the four ripening stages of fruits, (Musa AAA ‘Minai No. 1’), mainly involving M. acuminata (A genome). The total RNA from four different mature stages of three biological replicates was extracted using the Trizol (Invitrogen, Carlsbad, CA, USA) kit. A total of 12 cDNA libraries were constructed using Illumina’s NEBNext^®^ UltraTMRNALibraryPrepKit (New England Biolabs, Ipswich, MA, USA), and after the quality control was completed, |log_2_FoldChange| ≥ 1 and FDR < 0.05 were used as the screening conditions for DEGs. In this experiment, the expression data of MaXTH genes were extracted from the above-mentioned transcriptome, converted by log_2_ (FPKM + 1), and represented by FPKM values. TBtoolsv2.309 was used to draw the expression heatmap [31] to analyze the expression of MaXTH family members and screen out the highly expressed genes.

### 4.4. Analysis of the XTH Pan-Gene Family in the Musa Genus

Genome data of the 16 species were downloaded from the banana genome hub website (https://banana-genome-hub.southgreen.fr/, accessed on 1 November 2025). Using the *Arabidopsis thaliana* XTH family member accession number [32], the XTH gene family protein sequences were downloaded from the arabidopsis genome database (TAIR, http://arabidopsis.org/, accessed on 1 November 2023). The bioinformatics software TBtools was utilized to perform a blast search (E-value < 1 × 10^−5^) of the protein sequences of the 16 bananas against the arabidopsis XTH protein sequences. The XTH Hidden Markov Model (HMM) was downloaded from the Pfam website (PF00722 and PF06955) and used to conduct a search to obtain XTH family members. By screening through these two methods, preliminary candidate members were obtained. The domain structure was validated using the CDD website (https://www.ncbi.nlm.nih.gov/cdd, accessed on 1 November 2023) [33], and sequences that do not contain Glyco_hydro_16 and XET_C were removed to obtain the final members. The banana XTH members were named by referring to the nomenclature of *XTH* genes in *Arabidopsis thaliana*. The *Musa* species accessions analyzed and their genome types are shown in Supplementary Table S3.

The bioinformatics software TBtools was used to predict the function of the XTH family in the *Musa* genus. In addition, the online websites CELLO (http://cello.life.nctu.edu.tw/, accessed on 1 November 2023) and SignalP 6.0 Server (https://services.healthtech.dtu.dk/services/SignalP-6.0/, accessed on 1 November 2023) were used, respectively, to predict the subcellular localization and signal peptides of the proteins of the XTH family in the *Musa* genus.

TBtools software was used to analyze the gene structure and conserved motifs of the XTH family in the *Musa* genus. The CDD online website (https://www.ncbi.nlm.nih.gov/cdd, accessed on 1 November 2023) was used to conduct an analysis of the conserved domains, and TBtools software was used to visualize the results.

The MaXTH protein sequences were extracted from the banana genome database and subjected to blast comparison analysis with the protein sequences of 17 species with high homology (*Solanum lycopersicum*, *Arabidopsis thaliana*, *Nicotiana tabacum*, *Medicago truncatula*, *Zingiber officinale*, *Oryza sativa*, *Triticum aestivum*, *Hordeum vulgare*, *Ginkgo biloba*, *Cycas* spp., *Pinus lambertiana*, *Pinus taeda*, *Gnetum montanum*, *Zygnema*, *Chara braurii* Gmel, *Physcomitrella patens*, and *Selaginella moellendorffii Hieron*). The MEGA7 software was used to construct a phylogenetic tree (https://www.megasoftware.net/, accessed on 1 November 2023) [34]. To further analyze the structural characteristics of MaXTH proteins, SOMPA software (https://npsa-prabi.ibcp.fr/cgi-bin/secpred_sopma.pl, accessed on 1 November 2023) was used to predict the secondary structures of the XTH family proteins; the online software SWISS MODEL (https://swissmodel.expas-y.org/interactive, accessed on 1 November 2023) was used to predict the tertiary structures of the XTH family proteins. STRING online software (https://cn.string-db.org/, accessed on 1 November 2023) was used to predict the protein–protein interaction relationships among the members of the XTH family.

### 4.5. Chromosomal Localization, Promoter Analysis, and Gene Duplication Analysis of the XTH Gene Family in the Musa Genus

TBtools software was used for chromosomal localization analysis and visualization of the XTH family in the *Musa* genus. Taking the 2000 bp upstream of the XTHs in the *Musa* genus as the promoter sequence, the PlantCARE online website (https://bioinformatics.psb.ugent.be/webtools/plantcare/html, accessed on 1 November 2023/) was utilized to predict the promoter characteristics and the features of *cis*-acting elements of different members. Excel was used for statistical analysis, and the results were visualized. TBtoolsv2.309 software was employed to analyze the intragenomic collinearity relationships of the XTH genomes of 16 *Musa* species accessions. Referring to the method of Chengjie Chen et al. [31], TBtools software was used to visualize these relationships.

### 4.6. Subcellular Localization Analysis, and Banana Transient Transformation

The full-length coding sequences of the *MaXTH32.5* without stop codons were amplified using primers (Supplementary Table S1) and cloned into the pCAMBIA1302-35S-GFP vector via the KpnI and BamHI restriction sites. PCAMBIA1302-35S-*MaXTH32.5*:GFP was transiently expressed in tobacco (*N. benthamiana*).

The successfully constructed plasmid pCAMBIA1302-*MaXTH32.5*-GFP and the empty vector were each introduced into Agrobacterium strain EHA105, and banana fruits were transformed by injection with the bacterial suspensions [35]. After inoculation, the fruits were incubated at 25 °C for 3 d, and fruit firmness was then measured with a firmness tester.

### 4.7. Quantitative Real-Time PCR Analysis

The Primer3 online website (https://bioinfo.ut.ee/primer3-0.4.0/, accessed on 1 November 2023) was used to design RT-qPCR primers for the screened genes (Supplementary Table S1), and these primers were synthesized by Beijing Tsingke Biotechnology Co., Ltd. (Beijing, China). The total RNA of fruits in four ripening stages and transient transformation banana fruits were extracted using the plant total RNA extraction kit, which was provided by Tiangen Biotech (Beijing, China) Co., Ltd. The total RNA was reverse-transcribed into cDNA using the RevertAid Master Mix Kit, which was provided by Thermo Fisher Scientific (Waltham, MA, USA) Co., Ltd. Taking the Clathrin Adaptor Complexes Medium Subunit Gene (*CAC*) as the internal reference gene [35], the Roche Light Cycler 96 fluorescence quantitative PCR instrument was used to detect the expression levels of *XTH* genes in (*Musa* AAA) fruits in four ripening stages. Excel software was used to process the data, and the 2^−∆∆CT^ method was used to calculate the relative gene expression levels. GraphPad Prism9.5 software was used to analyze and visualize the data (GraphPad Software, San Diego, CA, USA, www.graphpad.com). One-way analysis of variance (ANOVA) and analysis of significant differences (*p* < 0.05) were performed on the results, and bar charts were generated.

## 5. Conclusions

During the ripening of banana fruits, fruit firmness continuously decreases, starch content fluctuates with an initial increase followed by a decrease, and soluble protein content rises steadily, and RNA-seq analysis of 12 cDNA libraries from four ripening stages (S1–S4) identified ripening-related DEGs enriched in protein processing and degradation pathways, with 5 MaXTHs up-regulated and 4 down-regulated in S2–S4 versus S1. The RT-qPCR results validated this finding. A total of 989 XTH family members were identified in the *Musa* genus, and comprehensive predictive analyses were performed on their physicochemical properties, gene structures, conserved motifs/domains, chromosomal localizations, and other characteristics. The results indicated that XTHs are evolutionarily conserved with functional diversity. *MaXTH32.5* was localized at the chloroplast, and transient transformation of *MaXTH32.5* affects banana fruit firmness and may be involved in regulating ripening in banana fruits. This research provides a foundation for future studies such as gene editing and protein function verification of XTHs.

## Data Availability

Data are contained within this article and its Supplementary Materials. Raw (unprocessed) data are available on request from the corresponding authors.

## Supplementary Materials

The following supporting information can be downloaded at https://www.mdpi.com/article/10.3390/plants14243810/s1. Table S1: The primer of PCR and RT-qPCR. Table S2: The basic properties of the XTH32.5 gene family. Table S3: Musa species accessions analyzed and their genome types. Figure S1: The DEG volcanic map in the S1 vs. S2, S1 vs. S3, and S1 vs. S4 comparison. Figure S2: The RT-qPCR in different banana ripening stages. Figure S3: The phylogenetic tree of the 16 *Musa* species accessions.

## Abbreviations

The following abbreviations are used in this manuscript:

d	Day
DEGs	Differentially Expressed Genes
EV	Empty Vector
GO	Gene Ontology
KEGG	Kyoto Encyclopedia of Genes and Genomes
OE	Overexpression
RT-qPCR	Quantitative Real-Time Polymerase Chain Reaction
VS.	versus
XTH	Xyloglucan endotransglucosylase/hydrolase
