# Supplementary figures and images for: Transcriptomics and Gene Family Identification of Cell Wall-Related Differentially Expressed Genes Reveal MaXTH32.5 Involved in Fruit Firmness During Banana Ripening
Source: Plants (Basel). 2025 Dec 14;14(24):3810. doi: 10.3390/plants14243810 (PMC12736723; doi:10.3390/plants14243810)

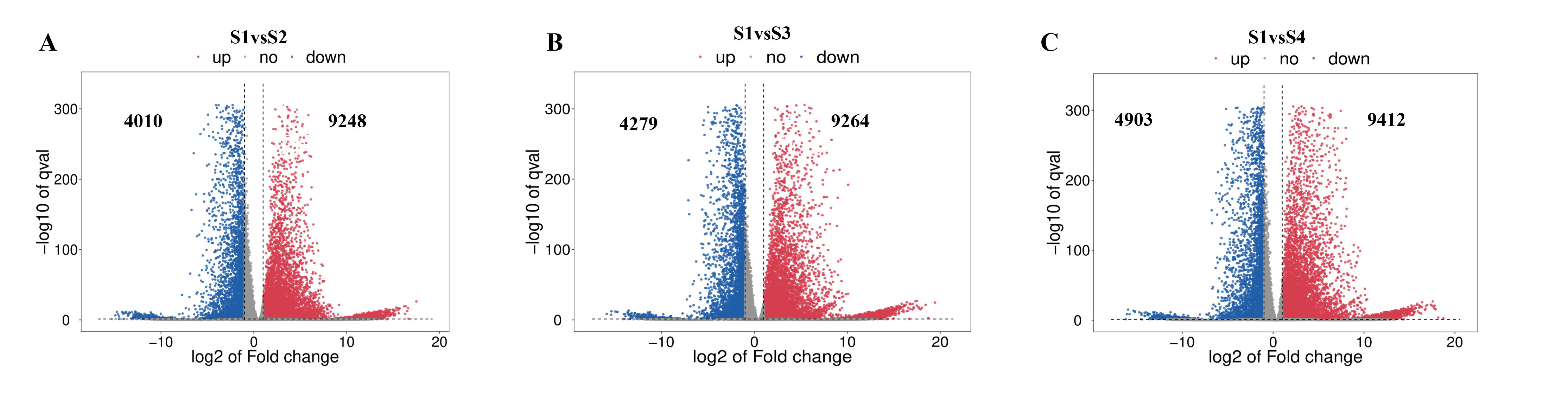

Supplement: Supplementary file 1 [file plants-14-03810-s001.zip › Suppl. Fig. 1.tif]

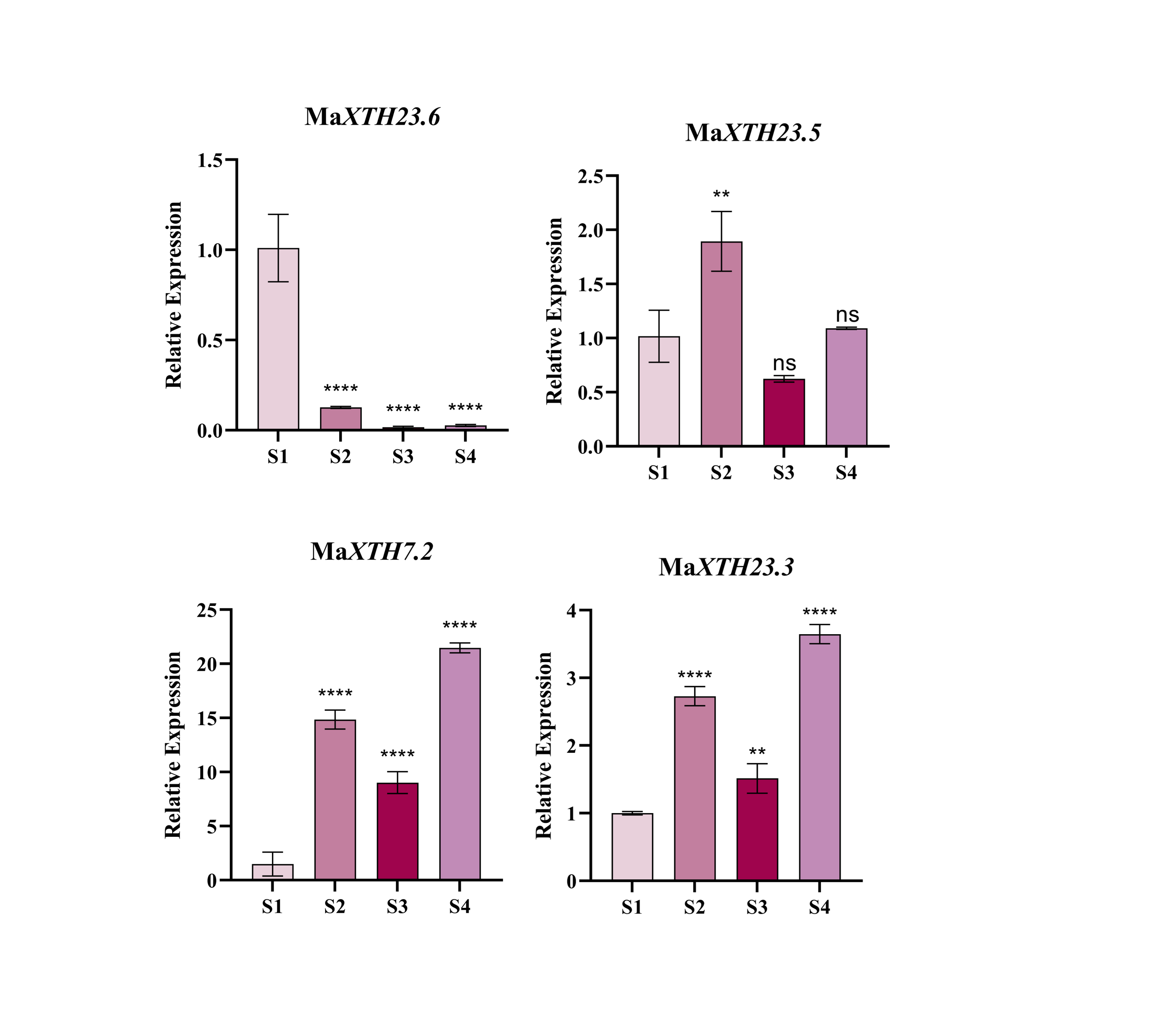

Supplement: Supplementary file 1 [file plants-14-03810-s001.zip › Suppl. Fig. 2.tif]
